# Composites and Copolymers Containing Redox-Active Molecules and Intrinsically Conducting Polymers as Active Masses for Supercapacitor Electrodes—An Introduction †

**DOI:** 10.3390/polym12081835

**Published:** 2020-08-16

**Authors:** Rudolf Holze

**Affiliations:** 1 Institut für Chemie, AG Elektrochemie, Chemnitz University of Technology, D-09107 Chemnitz, Germany; rudolf.holze@chemie.tu-chemnitz.de; 2 Institute of Chemistry, Saint Petersburg State University, St. Petersburg 199034, Russia; 3 State Key Laboratory of Materials-oriented Chemical Engineering, School of Energy Science and Engineering, Nanjing Tech University, Nanjing 211816, China

**Keywords:** intrinsically conducting polymers, copolymers, supercapacitors, electrochemical energy storage

## Abstract

In this introductory report, composites and copolymers combining intrinsically conducting polymers and redox-active organic molecules, suggested as active masses without additional binder and conducting agents for supercapacitor electrodes, possibly using the advantageous properties of both constituents, are presented. A brief overview of the few reported examples of the use of such copolymers, composites, and comparable combinations of organic molecules and carbon supports is given. For comparison a few related reports on similar materials without intrinsically conducting polymers are included.

## 1. Introduction

Supercapacitors have been established as a means of electrical energy storage in a vast range of sizes, from tiny ones in environmental and medical applications across numerous mobile applications in, e.g., telecommunication, to large ones in electric vehicles in mass transit. Introductory overviews are available [1,2,3], and in numerous monographs presumably all aspects from basic functional principles to materials and applications are treated [4,5,6,7,8,9]. Initially, carbon-based electrode materials were used in preference, using the electrochemical double layer capacity for charge and thus energy storage (EDLC supercapacitors). Tremendous power densities have been achieved, while the energy densities achieved are still disappointingly low. The discovery of the pseudocapacitive behavior of many electrode/electrolyte solution interfaces [10], suggesting electrode materials with storage capabilities one to two orders of magnitude larger with only slightly diminished current capabilities, seemed to provide relief and an avenue to supercapacitors with higher energy density that was closer to the merger of batteries and supercapacitors [11,12,13,14]. Unfortunately, some of the numerous metal oxides and chalcogenides examined so far show insufficient stability, with mechanisms of deterioration potentially including recrystallization, Ostwald ripening, loss of active surface area, passivation, dissolution or phase transformation, which are not yet sufficiently understood [15].

Intrinsically conducting polymers (ICPs), suggested as active materials for secondary batteries almost immediately after the discovery of their redox behavior (see e.g., [16,17,18,19,20,21]), were subsequently proposed as active masses for supercapacitors, too [22,23]. Their use as active mass or as an additional ingredient improving stability and other performance data has been reviewed elsewhere [24,25,26]. One particularly attractive option appears to be the combination of an ICP with a metal chalcogenide, yielding a composite with performance data significantly superior to the respective data of the single components [27,28]. The actual function of the ICP in such composites seems to be unresolved in most cases.

**Figure 1:**
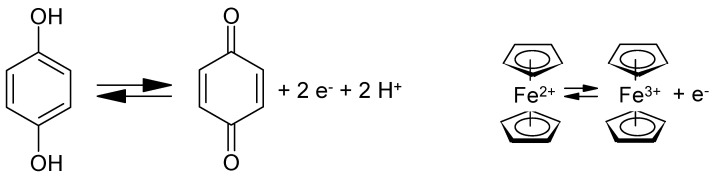
Schematic redox reactions of quinone and of ferrocene.

The recent surge of interest in organic materials (monomers, oligomers, polymers) as active masses in electrodes of secondary batteries [29,30,31,32,33] has moved beyond the developments briefly outlined above; more recently, interest in possible use in supercapacitor electrodes has been added. In addition to surpassing the inherent limitations of currently used materials, reductions in cost and the reduction of toxic or otherwise limited cell inventories have been added as aims, as demonstrated by using, e.g., anthraquinone-modified carbon as a negative electrode with RuO_2_ in the positive electrode of an asymmetric supercapacitor, saving 64% of the otherwise necessary ruthenium [34]. Studies of molecules or repeat units in larger entities showing a defined redox activity that can be employed for charge storage and when combined into a cell or capacitor for energy storage have focused so far on molecules related to quinone and on ferrocene-related species (see Figure 1).

The interest in the former is also due to the possible use of natural sources like lignin containing significant amounts of quinone-like units [35]. Reports do not necessarily suggest a particular use for either battery or capacitor; this is obviously in line with the merger of the two principles and the associated technologies already addressed above [11]. Further use in, e.g., redox flow batteries has been addressed elsewhere [36].

**Figure 2:**
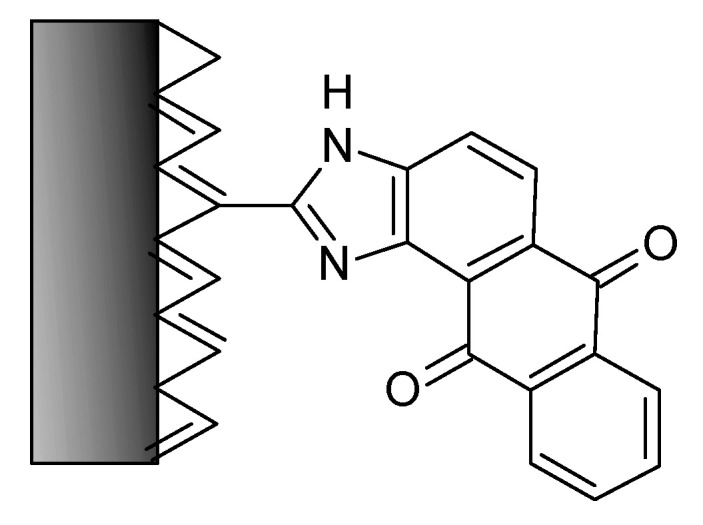
Proposed structure of 1,2-diaminoanthraquinone attached to a carbon surface.

The redox capabilities of organic molecules can be employed in different ways. The material of interest can simply be mixed with a binder and, if needed, a conductive material like acetylene black. The molecules can be attached by simple chemical procedures to carbon supports, as demonstrated with an asymmetric supercapacitor employing a negative electrode modified with anthraquinone AQ and a positive electrode modified with 1,2-dihydroxbenzene, which yielded a doubled energy density of the complete device [37]; for details on the modification with AQ, see [38]. An even simpler approach to chemical linking resulting in a proposed structured depicted in Figure 2 has been proposed, but has apparently not been examined with respect to application in a supercapacitor electrode [39].

Some relevant monomers, like 1-amino-anthraquinone can be electropolymerized onto an electronically conducting substrate like carbon [40,41,42,43,44]; because of electrode potential-dependent electron conduction in the polymer generated this way in the polymer during redox transformations, charge trapping may be observed [45].

**Figure 3:**
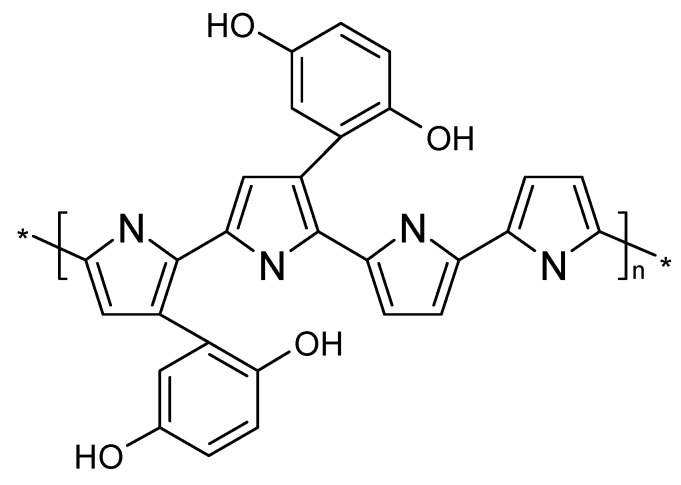
ICP chain with pendant quinone redox moieties (simplified scheme).

**Figure 4:**
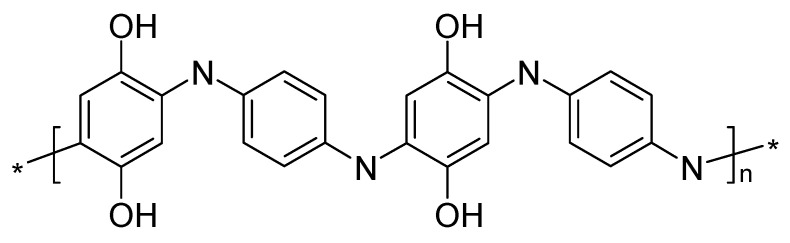
ICP with redox moieties incorporated in the molecular chain (simplified scheme).

The molecules can also be made part of a copolymer involving, for example, an intrinsically conducting polymer and said redox-active entity. Two fundamentally different approaches with implications for the molecular structure, the starting materials and the preparation process are conceivable: the redox-active moieties are attached to the molecular backbone of the conducting polymer (Figure 3), or can be made part of the copolymer chain (Figure 4).

Both products are true copolymers; for details of nomenclature and experimental approaches towards a distinction, as well as differentiation from polymer blends or alloys or simple mixtures of homopolymers, see [46]. With respect to optimized electronic conductance of the ICP-component, a structure as suggested in Figure 3 seems to be advantageous because the conjugation along the chain necessary for charge movement (i.e., conduction) along the molecular chain is not disturbed, or potentially interrupted, by different molecules like quinone-units in a polyaniline chain. 

A further option not based on covalent linkage is provided by the use of functionalized quinones like anthraquinone-2-sulfonate as dopant anion with an ICP [47]. As the AQ salt is substantially soluble in water anion exchange between anions attached to the polymer chain and free anions in the electrolyte solution may cause losses of storage capability and/or self-discharge. Possible means of keeping anions in place, like using gelled electrolyte solutions, may become helpful. An actual appraisal of conductance, its change of electrode potential (state of charge of the electrode material) and stability will become available only with actual samples.

## 2. Quinones in Supercapacitors

Lignin, as a widely available biopolymer of biological origin, was combined with PEDOT by Navarro-Suárez et al. into an electrode material using the quinone/hydroquinone moieties in lignin [48]. Beneficial effects of the presence of PEDOT were noticed, a somewhat confusing notation leaves the question open as to whether the quinone/hydroquinone moieties provided redox storage (most likely), or capacitive storage as stated by the authors.

Ren et al. polymerized chemically aniline in a solution also containing *p*-benzoquinone and reduced graphene oxide [49] (the title of the report claims graphene, apparently the authors assume graphene and rGO to be the same). Formation of polyhydroquinone was claimed, but not substantiated. As compared to a material containing only PANI, a slightly diminished storage capability but vastly enhanced stability with 94% capacitance retention after 1000 cycles was found. The stated layered structure of the product remains unclear. Oxidation of aniline with *p*-benzoquinone yielded PANI with some 2,5-dianilino-*p*-benzoquinone as a byproduct [50]. Performance as an electrode material was not examined.

A supercapacitor employing two PANI-modified electrodes soaked with quinone, as well as hydroquinone employing an aqueous electrolyte solution containing sulfuric acid has been described by Vonlanthen et al. [51]. A glass filter disc (thickness 2 mm), permeable for both ions and molecules (i.e., a separator) kept the electrolyte solutions of both half-cells from mixing, thus reducing self-discharge to an acceptable level. This aspect—presumably a major limitation of this concept—is nowhere addressed in the report. The observed high electrocatalytic activity of PANI for the BQ/HQ-redox couple has been reported much earlier [52,53,54]. Capacitances were stable up to 50,000 cycles.

Self-assembled nanostructures of graphene and anthraquinone-2-sulfonate have shown various advantages: the anions keep the graphene from restacking by interactions between the anions and the graphene and provide redox capacitance [55]. After 100,000 cycles, 82% of the initial capacitance was still present.

PPy with anthraquinone sulfonate as counteranion deposited on activated carbon cloth in the presence of reduced graphene oxide yielded an electrode material with 90% capacitance retention after only 100 cycles [56]. Replacing perchlorate dopant ions in PPy with anthraquinone sulfonate as reported by Wan et al. [57] provided a substantial increase in storage capability and stability, after 1000 cycles, about 90% of the initial capacitance were left. Yoneyama et al. used anthraquinone-1-sulfonate as dopant anion with PPy [58]. This addition doubled the charge storage capability of the ICP. Without data on capacitance retention with cycle number the claimed fixation of the anions in the ICP can hardly be judged. Various anthraquinone sulfonates have been suggested as counter anions in PPy [59]. A changed morphology of the polymer presumably related to the presence of the anthraquinonesulfonates during synthesis. The highest specific capacitance was observed with 9,10-anthraquinone-2-sulfonate, the value increased by about 150% when compared with pristine PPy using perchlorate ions. Beyond the effect of the changed morphology resulting obviously in a larger EASA, the redox process of the anthraquinonesulfonates (for further details see [34,38]) contribute to charge storage.

Simple adsorption (the experimental procedure suggests deposition with the actual mode of interaction between AQ and the substrate left open. The notoriously poor solubility of AQ in aqueous solutions might just keep the AQ in place without any further interactions) of AQ on chemically functionalized single wall carbon nanotubes yielded an electrode material showing two redox transitions for the strongly adsorbed AQ different from the frequently observed single peak [60]. Initially the authors assumed two subsequent electron transfers with the material studied here, but the strikingly different charges of the observed peaks ruled this out easily. Instead, adsorption of AQ on energetically different sites was invoked as an explanation; obviously, such different sites were not noticed in previous studies. Use as a supercapacitor electrode material has not been examined.

The use of quinones grafted onto carbon supports has been reviewed selectively [61]. Limitation by accelerated self-discharge observed with quinones added to the electrolyte solution (somewhat contradicting the title of this report) has been highlighted.

**Figure 5:**
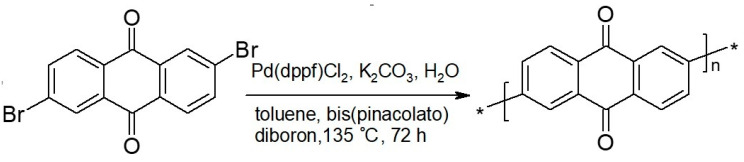
Schematic synthetic route to polyanthraquinone [63].

Wang and Feng electropolymerized AQ onto graphite felt for use as an electrode in a microbial fuel cell [62]. The obtained electrode showed battery electrode-like behavior. Zhou et al. prepared polyanthraquinone via a chemical synthesis route starting with 2,6-dibromoanthraquinone (see Figure 5) in the presence of Ketjenblack (a carbon black) [63].

Supercapacitive performance of the mass blended with conducting carbon and PTFE as a binder was assessed in a nonaqueous electrolyte solution. The observed CV showed a redox current peak pair assigned to the quinone/hydroquinone reaction. Combination of the polymer with Ketjenblack improved stability significantly, 85% capacitance retention was observed after 1000 cycles.

Catechol has been grafted on Black Pearls activated carbon using diazonium chemistry [64]. Loss of capacitance observed over 10,000 cycles was attributed mainly to loss of catechol redox activity. Using impedance measurements, the decreased high current rate capability of the catechol-grafted material was confirmed [65].

**Figure 6:**
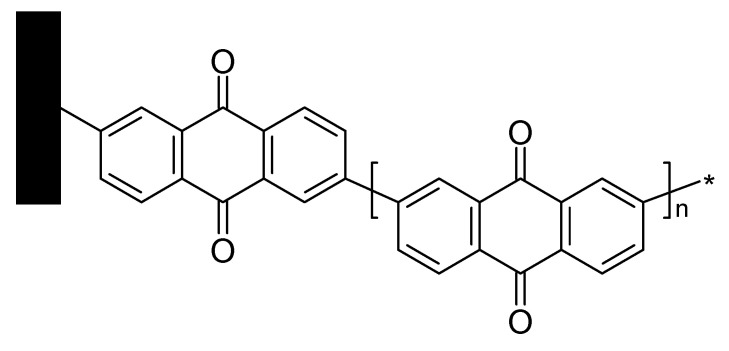
AQ grafted onto carbon cloth [66].

2-Aminoanthraquinone has been grafted onto carbon cloth using said chemistry resulting in a 2.5-fold increase of overall interfacial capacitance [66]. Formation of chain-like structures attached to the cloth was suggested (see Figure 6).

The major increase in capacitance caused by the attached quinone moieties shows as battery–electrode-like behavior of the obtained material evidenced in the galvanostatic charge/discharge curves. 58% of the initial capacitance was observed after 10,000 cycles. The influence of electrolyte solution pH on the behavior and performance of AQ grafted on carbon with two different precursors (1- and 2-diazoanthraquinone) has been examined [67]. Use of the attached quinone moieties was found to depend on pH of the electrolyte solution. 17% capacitance loss after 10,000 cycles was reported for this electrode material [68]. The amount of actually grafted AQ was determined using 1-amino,5-chloroanthraquinone as an example [69,70]. The amounts determined with elemental analysis and further spectroscopic techniques of the chlorine content and from slow scan CV agreed well. An alternative procedure for grafting AQ avoiding diazonium chemistry, and thus claiming to be “simpler and greener”, has been proposed [71]. It involves either spontaneous grafting (the procedure involves use of NaNO_2_, it thus may be not that ecofriendly nor spontaneous) in acidic electrolyte solution or electrochemically induced grafting. The measured storage capability was similar to the one observed with electrode materials prepared in the traditional, i.e., diazonium chemistry, way. Cycling stability was higher. The electrochemical procedure enhanced grafting efficiency and reduced the amount of AQ left unattached in the carbon. This fraction might desorb during electrode operation possibly causing increased self-discharge.

**Figure 7:**
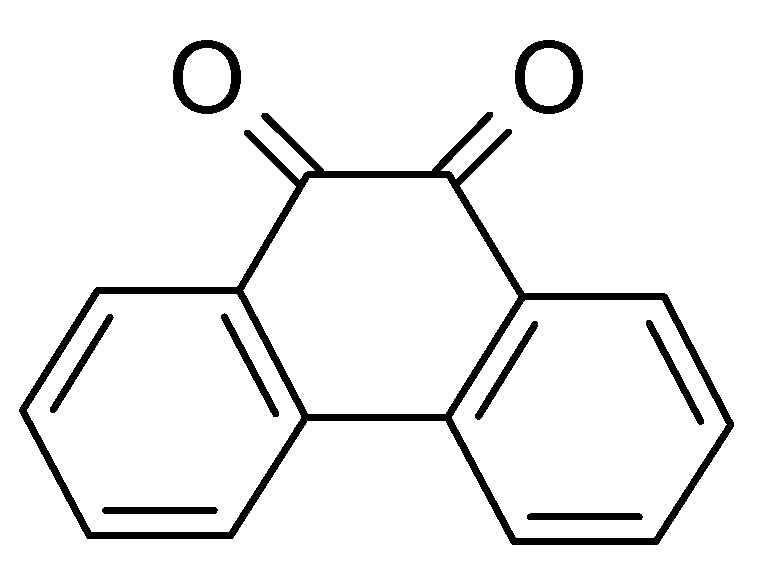
9,10-phenanthrenequinone.

Grafting 9,10-phenanthrenequinone (see Figure 7) onto porous carbon yielded a negative supercapacitor electrode with 76% capacitance retention after 10000 cycles at 11 wt% loading of the carbon with the quinone [72,73]. In a full device, the additional quinone resulted in 1.4-fold increase of specific energy. At 20 wt%, this figure was 2.5.

**Figure 8:**
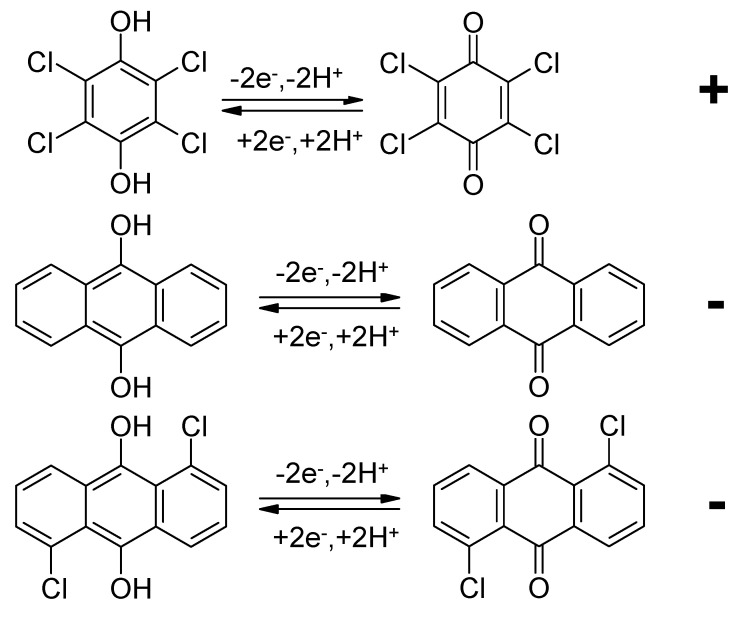
Redox reactions of tetrachlorohydroquinone (**top**), AQ (**middle**) and 1,5-dichloroanthraquinone (**bottom**).

Activated carbon impregnated (by adsorption) with tetrachlorohydroquinone at the positive electrode and AQ or 1,5-dichloroanthraquinone (see Figure 8) at the negative electrode has been examined in supercapacitor cells [74].

Activated carbon modified with various quinones by sublimation showed enhanced capacitances, plain AQ yielded the highest increase [75].

**Figure 9:**
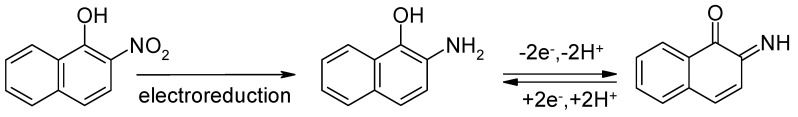
Electrochemical transformation of 2-nitro-1-naphthol into 2-amino-1-naphthol and redox electrochemistry of the latter.

Simple blends of AQ or further substituted quinones with porous carbon materials have been suggested as electrode materials [76], another option is ordered mesoporous carbon modified with AQ [77]. Quinone decorated onto MnO_2_ provided a significant increase of capacitance at 93% capacitance retention after 300 cycles [78]. Carbon electrodes have been modified with 2-nitro-1-naphthol by simple adsorption from a mixture of water and methanol [79]. After initial electroreduction of 2-nitro-1-naphthol into 2-amino-1-naphthol subsequently in charge/discharge of the electrode the quinone/hydroquinone redox reaction was employed for additional charge storage beyond the capacitive storage at the electrolyte solution/carbon interface (Figure 9).

**Figure 10:**
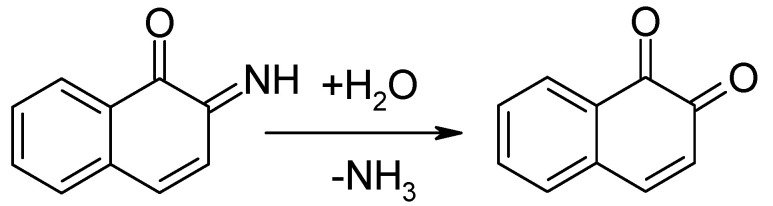
Hydrolysis of *o*-naphthaquinoneimine into 1,2-naphthaquinone.

The redox system using 2-amino-1-naphthol (*o*-aminonaphthol) and the *o*-naphthaquinoneimine is chemically not stable and hydrolyzes yielding 1,2-naphthaquinone (Figure 10).

**Figure 11:**
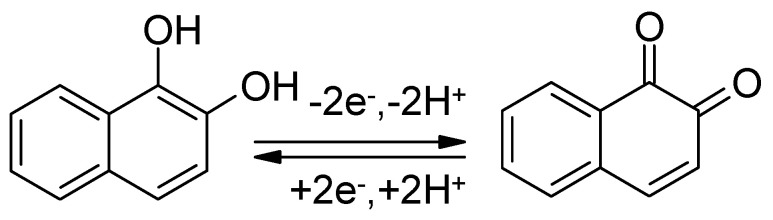
1,2-naphthaquinone/1,2-naphthahydroquinone redox couple.

Subsequently, the 1,2-naphthaquinone/1,2-naphthahydroquinone redox couple (Figure 11) enables charge storage:

During 1000 cycles, about 20% of this redox capacity was lost.

The use of substituted AQs (e.g., brominated AQ) [80,81] and of hydroquinone and its isomers [82,83,84] as redox-active additives in electrolyte solutions for supercapacitors has been reported. Because of the presumably rapid self-discharge—data on this practically highly relevant aspect are hardly reported—this approach appears to be of limited technical value with an undivided cell. Insertion of a separator will increase the Ohmic cell resistance making high current capability unlikely. Self-discharge of both single electrodes and complete two-electrode cells using quinones and AQ grafted onto carbon as redox active constituent or using dissolved quinines has been examined [85]. Hydroquinone added into the electrolyte solution for increased storage capability results rather expectedly in much faster self-discharge (50% loss of cell voltage in 0.6 h) than quinones covalently attached to an electrode (50% loss of cell voltage in 6 h). A higher use of the AQ covalently attached to the electrode by about three orders of magnitude is noticed. This once again is not unexpected given the presumably overwhelmingly large fraction of dissolved quinone located anywhere in the electrolyte solution far away from the electrode. Addition of dibromodihydroxybenzene to the alkaline electrolyte solution of an EDLC system resulted in an increase of recorded capacitance; this was also observed when quinone and potassium bromide were added [80]. Because of the numerous reaction pathways accessible for the added species the actual redox processes responsible for charge storage remain open.

A composite of graphene and PPy has been prepared using either anthraquinone-2-sulfonate or anthraxquinone-2,6-disulfonate as oxidants during chemical polymerization [86]. The AQs remained in the product providing additional storage capacity up to 100% more when compared with the composite containing no AQ or a composite of graphene and AQ.

## 3. Ferrocenes in Supercapacitors

**Figure 12:**
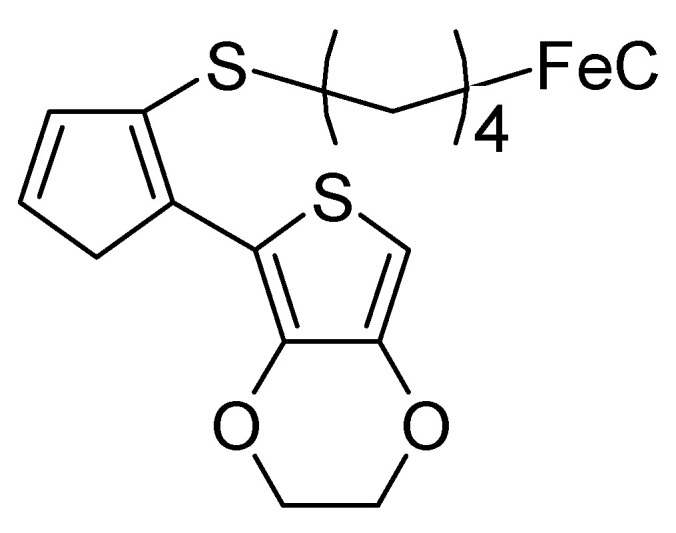
Substituted bithiophene as used by Peng et al. [87].

Peng et al. [87] electrosynthesized an ICP from a monomer [88], as depicted in Figure 12. 

From CVs at different electrode potential windows, a charge transfer of up to 0.93 electrons per repeat unit (see Figure 12) was determined; in addition, separation of the redox and the double layer charge current was possible. With the widest window, two redox peak pairs assigned to the redox process involving the ferrocene couple (the far larger peak) and the repeat unit in the ICP backbone (the much smaller peak) could be distinguished. In this report, once more the contributions of both redox processes and electrochemical double layer are stressed, independent determination of the number of electrons per repeat unit and double layer charge are recommended.

Li and Zheng copolymerized chemically aniline and 1,1-ferrocenediacyl anilide [89,90] with ferrocene units in the molecular chain. Displayed CVs seem to suggest only minor contributions of redox activity from the ferrocene unit; the copolymer combined with reduced graphene oxide rGO in turn provided only minor charge storage contributions. Charge/discharge curves indicate otherwise. Assuming that similar electrode masses were employed, the composite provided doubled storage capability. The obviously erroneous labeling in the picture casts doubt on this conclusion. The higher ESR (electrical series resistance) of the copolymer as compared to the rGO was reduced in the composite to a value even lower than for rGO. This was attributed to covalent linkages between the copolymer and rGO not proven in the report. More general considerations on graphene functionalized with ferrocene and other organometallic compounds have been reported [91]. A PANI-stabilized composite of ferrocene and graphene has been prepared showing 86% capacitance retention after 5000 cycles [92]. Graphene quantum dots chemically modified with ferrocene and combined with PPy into an electrode material kept 86% of the initial capacitance after 5000 cycles [93].

A nanostructured hybrid of polyvinylferrocene and PPy with the latter acting as a “molecular wire” ameliorating the poor electronic conductivity of the non-conjugated polyvinylferrocene has been prepared [94]; after 3000 cycles, 94.5% of the initial capacitance was still present. Ferrocenated gold nanoparticles have been used to modify porous carbon, the obtained material has been suggested as an electrode material for supercapacitors [95].

**Figure 13:**
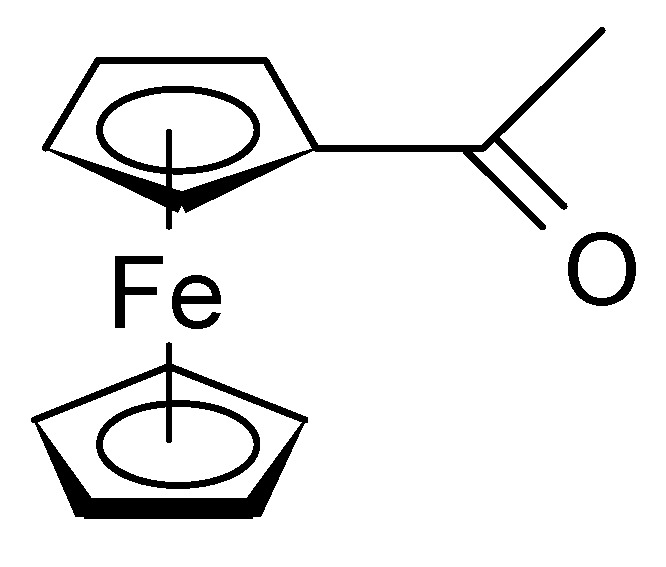
Ferrocene-CHO.

GO has been functionalized with ferrocene-CHO (Figure 13) and with porphyrin-CHO [91].

**Figure 14:**
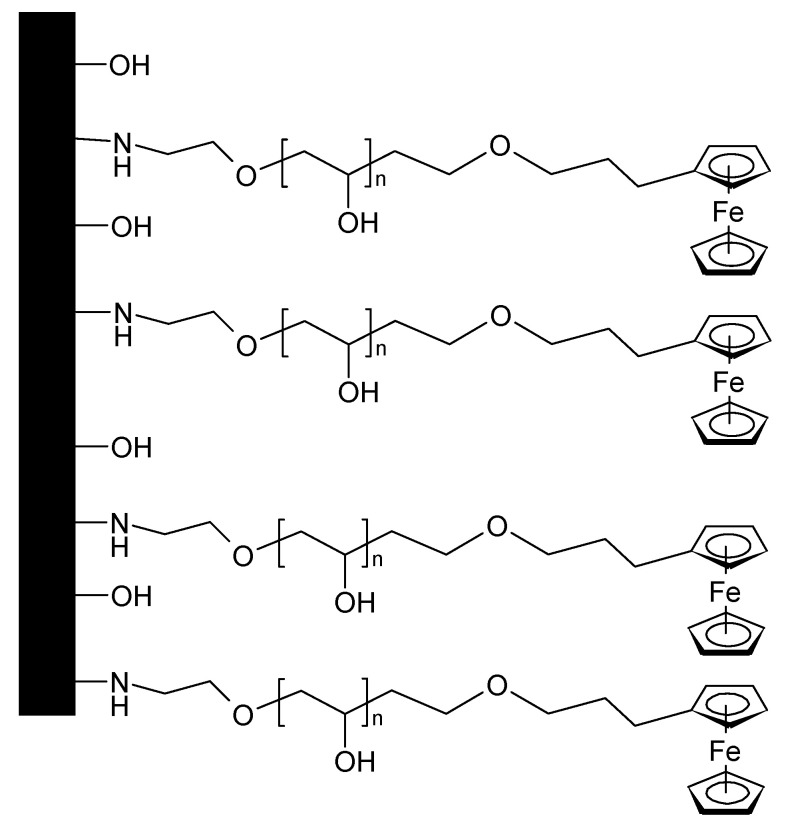
Ferrocene grafted onto GO.

The ferrocene-modified material showed a very wide redox response compared to the well-defined redox-peak pair of molecular ferrocene. The mostly capacitive response of GO in a CV is slightly diminished with the ferrocene-modified GO, and the GCD-curves suggest a pseudocapacitive behavior of the ferrocene-modified material, suggesting a strong electronic interaction between the ferrocene units, presumably facilitated by the graphene layer. After 5000 cycles, 93% of the initial capacitance were found. Ferrocene has been grafted on GO by ring-opening polymerization (Figure 14) [96], yielding a supercapacitor electrode material with 96% capacitance retention after 2000 cycles.

**Figure 15:**
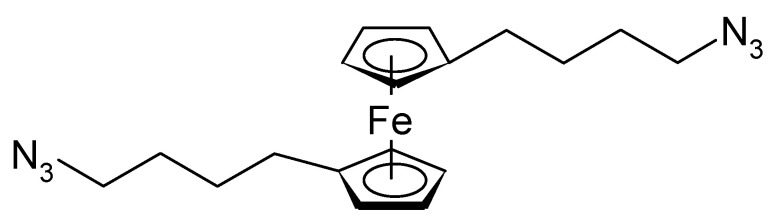
1,10-bis(4-azidobutyl)ferrocene [97].

1,10-bis(4-azidobutyl)ferrocene (Figure 15) has been attached with both acido-functions to reduced GO as a bridging moiety between sheets [97]. The electrode material kept about 80% of its initial capacitance after 2000 cycles.

**Figure 16:**
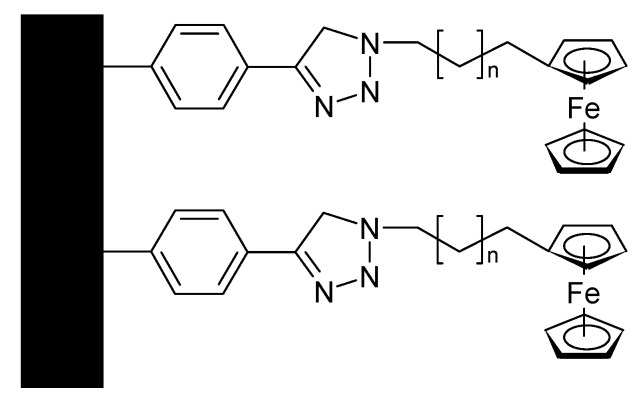
(11-azidoundecyl)ferrocene on carbon, n = 9.

4-Azidobutylferrocene (the singly substituted version of the compound shown in Figure 15) has been attached to rGO, with PANI a compound was prepared for use as a supercapacitor electrode material showing 89% capacitance retention after 2000 cycles [98]. A similar material with GO instead of rGO has been examined, too [99]. After 3000 cycles, 85.54% of the initial capacitance was retained. 2,2-bis(ethylferrocenyl)propane intercalated into GO and subsequently combined with PANI yielded an electrode material with 89.13% capacitance retention after 3000 cycles [100]. A monolayer of (11-azidoundecyl)ferrocene (Figure 16) on carbon has been examined with respect to electron transfer kinetics and possible application in a supercapacitor [101].

Qiu et al. functionalized carbon nanotubes with ferrocene [102]. The CVs do not show any response, possibly due to the FeC-units, just a rather asymmetric increase of a broad current response growing with increasing degree of functionalization. The changes were attributed to enhanced wettability of the CNTs. A H-π-interaction between ferrocene and graphene subsequently stabilized with PANI yielded a material that has been suggested as an active material for a supercapacitor electrode with 960 F·g^−1^ [92]. 5,10,15,20-tetraferrocenylporphyrin has been immobilized on CNTs; subsequently, this material was incorporated into a composite with copper nanoparticles [103]. 80% of the initial capacitance was retained after 3000 cycles.

A ternary composite of PANI, ferrocene-modified GO and Mn_3_O_4_ has been examined as an electrode material [104]; 20% capacitance loss after 3000 cycles was reported.

Further conducting materials like graphene and reduced graphene, as well as insulating materials like silica, can be functionalized with the redox active species of interest. Reduced graphene oxide functionalized with bis-acidoferrocene has been proposed as an active material for supercapacitor electrodes [96,97]. In an acidic electrode, an electrochemical response somewhere between pseudocapacitive and battery electrode-like with about 80% capacitance retention after 2000 cycles was recorded. Laser-reduced GO has been combined with ferrocene into a 3D composite showing 4% capacitance loss after 8000 cycles [105]. Spontaneous modification of surfaces of electrode materials by strong non-covalent attachment of a ferrocene-substituted pyrene initially dissolved in the electrolyte solution has been proposed as a means to increase storage capability of the electrode material [106].

Chitosan covalently modified with ferrocene combined with CNTs in a composite showed 99.93% capacitance retention after 2000 cycles as a supercapacitor electrode [107]. Nanosphere peptide assemblages of multifunctional tyrosine-ferrocene have been examined as supercapacitor electrode materials [108].

Beasley and Murray functionalized silica nanoparticles with ferrocene with about 600 ferrocene moieties per nanoparticle, this was about equivalent to a monolayer coverage [109]. No interaction between the ferrocene units was observed as might have expected given their closeness [110]. Redox charge storage capabilities were reported with respect to both dry mass and concentrated slurry.

Materials like polycatenated Co-MOF containing substituted ferrocene as building blocks [111] have been prepared, characterized and suggested as supercapacitor electrode material. Studies where ferrocene was used as a raw material to prepare specific porous carbon structures (for an example, see [112,113]) are beyond the scope of this introductory overview.

Redox-active species have been added to the electrolyte solutions in supercapacitors, mostly of the EDLC-type, in attempts to increase their storage capabilities. 1-ethyl-3-methylimidazolium ferrocenylsulfonyl-(trifluoromethylsulfonyl)-imide was studied by Wang et al. [114], and the charge storage mechanism was studied with NMR and dilatometry. At the negative electrode, the evidence suggested exclusively ion adsorption, whereas at the positive electrode, depending on the stage of charge, Faradaic processes were also observed. For another example, see [115].

The use of ferrocene reported, e.g., in the synthesis of CNTs [116] is beyond the scope of this report.

## 4. Conclusions

Feasibility and suitability of combinations of intrinsically conducting polymers and electrochemically redox-active molecules, in particular ferrocenes and quinones, as composites or copolymers as active masses for supercapacitor electrodes were examined. The results obtained and the examined materials seem in most cases to require further examination of stability with much larger cycle numbers than those reported so far—if reported at all. Participation of the employed components with respect to the active involvement of both redox-active molecules and intrinsically conducting polymers in charge storage seems to be in need of verification in many cases, better understanding might suggest approaches to optimization and further combinations.
